# Distribution and Reactivity of Myocrisin

**DOI:** 10.1155/MBD.1994.497

**Published:** 1994

**Authors:** W. Ewen Smith, John Reglinski

**Affiliations:** 1 Department of Pure and Applied Chemistry University of Strathclyde 295 Cathedral St. Glasgow G1 1XL United Kingdom


### DISTRIBUTION AND REACTIVITY OF MYOCRISIN

W. Ewen Smith and John Reglinski

Department of Pure and Applied Chemistry, University of Strathclyde, 295 Cathedral St., Glasgow G1 1XL, U.K.

### INTRODUCTION

For many years gold compounds have been used reluctantly by clinicians treating aggressive rheumatoid arthritis [1]. The reluctance stems from the fact that the compounds are effective only in some patients and from the potential toxicity of gold in vivo. Various alternatives have been suggested over the past 20 years and none have proved sufficiently good to completely replace gold as a major contributor in the therapy of rheumatoid arthritis. However, the in vivo chemistry of gold is extremely complex and it may be possible to improve the therapeutic effectiveness of gold compounds through an understanding of the chemistry. The discovery of an effective orally active gold compound - auranofin - led to comparative studies with the injectable drug myocrisin. One clear conclusion to be reached was that the distribution and in vivo reactivity of the two compounds was very different, suggesting that the optimum therapeutic-to-toxic ratio could be significantly improved by a better targetted compound. The purpose of the present article is to review work on the distribution and reactivity in vivo of gold following injection with myocrisin. Some current investigations into possible modes of toxic and therapeutic action are described.

### DISTRIBUTION OF MYOCRISIN

Myocrisin is usually given as an intermuscular injection once a week. Small samples of animal tissue taken from around the injection site show initially very high concentrations of gold which gradually reduce with time. Following the initial injection, most myocrisin diffuses to the plasma unchanged. However, within the first 12 hours, the majority becomes bound to the proteins leaving only a very small amount of unbound material [2]. The concentration of gold in each fraction varies somewhat depending on the separation technique used. Where gel permeation chromatography has been used [3], the gold is bound mainly to albumin with a smaller fraction bound to globulins. The main binding site in albumin is known to be one specific reactive thiol group, cysteine-34, to which gold complexes as gold(I) by an exchange reaction with the thiomalate ligand present in myocrisin [4]. Similar results are obtained on separation by electrophoresis [2] but the amount present in albumin is lower. In addition, in electrophoresis a small spot appears on the strip above the proteins. The position of this spot corresponds almost exactly to that of free myocrisin. The conclusion is that a small fraction of the gold present in albumin is removed as myocrisin by electrophoresis. It leads to the suggestion that some of the drug is loosely adsorbed onto the protein rather than complexed to a specific site. This is not surprising in that albumin can transport a range of exogenous molecules in hydrophobic or hydrophilic pockets.

A common therapeutic regime employed in myocrisin therapy is a 12 week course of once weekly injections of 50 mg of the drug. In such a treatment, the gold concentration in the plasma accumulates in a saw tooth pattern [2]. Immediately following injection there is an increase in the gold present in the blood stream. During the week, a significant fraction of this gold is either adsorbed into tissues or excreted leaving a smaller but appreciable fraction in the blood. Subsequent injections build on this base and raise the gold level in the plasma. Thus, in the 12 week period the gold concentration present in blood accumulates. The final concentration is usually between 2 and 3 μg per ml. This is regarded as an optimum level for therapy although there is no general agreement on the value of the gold concentration in plasma in determining effective treatment. Subsequently, a maintenance course of monthly injections is given to maintain an acceptable gold level over a longer period of time.

An attempt has been made to follow the gold concentration in 10 patients with time [2]. Since these patients attended an out-patient clinic, it was not always possible to obtain samples at the same time during therapy and all patients did not complete the course. The separation was carried out by electrophoresis and the gold analysed by carbon furnace atomic absorption spectrometry. The amount of gold removed from the plasma proteins by electrophoresis and believed to be free myocrisin and the gold γ-globulin : albumin ratio varied from patient to patient but in any one patient it did not change significantly during the 12 week period despite the increasing gold plasma concentration. These results suggest that there is no saturation of any one gold site and indicate the ready formation in vivo of a gold-globulin complex as well as a gold-albumin one. The fact that the distribution does not change much in any individual patient with increasing gold level is not surprising. The sulphydryl concentration in the plasma due principally to albumin is about 200-500 times larger than the gold concentration achieved during therapy. Thus, there can be no saturation of the albumin site. Albumin accounts for approximately half the protein mass in the plasma and γ-globulin is an appreciable fraction of the rest. As such, it is unlikely that any binding site on γ-globulin can be saturated at clinically relevant concentrations. It is possible that a protein present at lower concentrations does have a saturated site, but none have been identified so far and the presence of an excess of effective complexing sites would require very strong complexing for saturation to occur.

During the process of circulation and accumulation in plasma, an appreciable fraction of the gold transfers from plasma to the circulating cells [5]. The amount that transfers varies from patient to patient. Much of it is present in the cell membrane with smaller amounts in the cytosol. The amount in the cytosol varies. It is lower with myocrisin than with auranofin and it is higher in patients who smoke. Recent papers have highlighted the fact that smoking increases the amount of thiocyanate and cyanide in the blood stream [6]. It may be that gold cyanide forms and transports the gold across the membrane. Red cell gold concentrations do not appear to correlate with clinical improvement and although they are higher in smokers, there appears to be no added improvement in the disease among patients who are on myocrisin and who also smoke.

In the cytosol of red blood cells, appreciable fractions of the gold present binds to haemoglobin and glutathione but there are other high molecular weight fractions (see article by Tepperman and Elder [7]) suggesting that gold is bound to the functional active extrinsic and intrinsic membrane proteins such as the hexose transporter [8] and spectrin [7]. Efforts have been made to carry out SDS page separations on cell membrane derived from myocrisin treated erythrocytes. There is evidence of a new protein (~55kD) appearing following gold incubation [9]. Whether this results from a breakdown of an existing protein or is a genuine new species is not known.

In white cells, using electron microscopy with chemical analysis, gold deposits can be found around lysosomes and in small vesicles known as aurosomes [10]. These contain a high concentration of both gold and sulphur. These groups are the most obvious in electron micrographs and correspond to the highest concentrations of gold in specific parts of the cell. Gold has been postulated to work by preventing the release of lysosomal enzymes from the lysosomes in the cell and the grouping of these deposits in a suitable area of the cell is cited as evidence in favour if this mechanism. However, in cells, isolated vesicles containing metals can be used by the cell as a mechanism to neutralise toxic species and the gold in the aurosome may be in unreactive and polymeric in form. Thus the significance of the gold deposits observed is not clear. It could either have a therapeutic action or be a detoxification step.

The lack of any clear transport and delivery function for gold in vivo leads to its accumulation in most body tissues. High concentrations are obtained in such organs as kidney, liver and spleen as well as in bones [2]. Lesser quantities are found in hair, nails etc. One worrying feature of these gold distribution studies is that gold can be detected long after the cessation of myocrisin therapy. Why the gold is present for such long periods is not known but the likely explanation is that insoluble polymeric and unreactive gold(1) complexes or gold(0) colloidal particles are accumulated. Gold(1) complexes are usually favoured as the most likely storage form since the aurosomes found in the white cells contain high sulphur concentrations as well as high gold concentrations.

### REACTIONS OF GOLD COMPOUNDS

Myocrisin is a polymeric molecule known to occur with a number of different chain lengths [11], all of which react readily with thiols [12] in complex degradation reactions. These reactions are initiated by ligand substitution at the terminal thiolate gold bonds, which would appear to be less stable than the corresponding internal bridging thiolates. For example, the reaction between cysteine and myocrisin [2] has been studied by circular dichroism. It is clear from a kinetic study over a period of 2 days that a large number of intermediate species are formed. The final product of the reaction is gold(I) cysteine - an insoluble gold polymer. Prior to its formation, a very strong dichroism signal is obtained indicating the increasing rigidity of the species formed. Other thiols which have been tested and found to react include glutathione, and penicillamine [12].

The nature of the reactive site in albumin has been investigated in many studies. The main site being that present at cysteine 34. The question arises as to how many gold atoms remain on the sulphur. In essence, the polymeric myocrisin species has to enter a pocket on the albumin where the reaction occurs and there is no guarantee of complete breakdown of the polymer. Alternatively, the reaction involves transport of a fragment of the polymer obtained by substitution reactions analogous to these described above. It must be remembered that in-vivo albumin is present as a heterogeneous protein which comprises of three forms: the free sulphydryl form (cysteine 34), a mixed disulphide formed between cysteine 34 and cysteine and a mixed disulphide formed between cysteine 34 and glutathione all of which are potential sites for gold activity.

In vitro, in incubation studies of myocrisin and albumin, the total gold concentration in albumin rises to a maximum of one gold atom per available sulphydryl residue at cysteine 34 [13]. The amount of gold incorporated into the protein can be increased by the addition of glutathione to the gold albumin mixture. It is believed that the addition of glutathione to the mixture facilitates the reduction of either the glutathione mixed disulphide or more probably the cysteine mixed disulphide, and this leads to a higher than expected incorporation of gold in the protein. There is little evidence to support the formation of a second gold binding site which can bind gold strongly and it would seem that the loosely bound gold identified in the gel permeation [2] studies arises from absorption of unchanged myocrisin into a pocket in the protein. There could in addition be a weak association of the gold at other locations on the protein such as the copper or nickel binding site which contains a ligand set not entirely compatible with the soft metal ions.

That a significant amount of myocrisin quickly associates with albumin and that a significant fraction of the metal clears from the plasma pool between injections [2] has led to suggestions that in the therapeutic action of gold in rheumatoid arthritis, the albumin gold complex is somehow involved either in metal transportation or as a "buffer" by accepting gold in the plasma and releasing it in the synovial cavity. Although many studies can show efficient and stoichiometric incorporation of gold into albumin, the reverse reaction is more difficult and requires a ligand such as $CN^-$ or $RS^-$ which can penetrate into the pocket containing cysteine-34 and compete for the metal ion [14,15].

Although the reactions between albumin and myocrisin are relatively rapid and occur over a period of a few hours, it is more difficult to obtain an interaction between gold and γ-globulin. That this occurs in vivo is clearly evidenced by the distribution studies. In vitro, following incubation over a period of 24 hours, appreciable amounts of gold are found to be bound to this protein [13]. The amount of gold incorporated is postulated to achieve a value commensurate with one gold atom per protein molecule. However, in contrast to albumin, γ-globulin does not react with thiol specific reagents and shows no evidence of a free thiol group. Thus an analogous reaction to the interaction of myocrisin with cysteine 34 on albumin cannot be occurring. The reaction of myocrisin with γ-globulin and the effect of glutathione on albumin/myocrisin incubations prompted the question of whether myocrisin would react appreciably with disulphides in-vivo. In-vitro this was first shown using lipoic acid and Ellmans reagent [16]. The rate of reaction is slower than with thiols but is appreciable and varies significantly depending on the nature of the disulphide. This work has been considerably extended more recently, indicating that gold disulphide reactions are a distinct probability in in vivo situations. γ-Globulin has more reactive disulphides close to the swing region and reaction with these protein sites is already known for other species. Thus, it is postulated that the most likely source of reaction between γ-globulin and myocrisin is by reaction at these high energy disulphide sites. Preliminary experiments in this area indicate that the protein after prolonged incubation can be broken into smaller fractions [17]. This reaction itself is important and has been very little studied. The reason for its importance lies in the direct relationship between γ-globulin and immunological response. Antigens are polymerised globulins. A metal induced conformational change in γ-globulin as a consequence of gold therapy may directly involve myocrisin in immune regulation in arthritis.

The sulphydryl status of platelets is similar to that of the globulins in that they have no appreciable sulphydryl concentration on the outer surface as determined by Ellmans reagent. However, preliminary studies [18] using high levels of gold have shown that platelet activation is affected by myocrisin in-vitro. Due to the low myocrisin concentration administered clinically it is unlikely that this is a primary effect of gold in-vivo. However, it is likely that this action may be occurring via a disulphide interaction rather than via a thiolate interaction. It can be difficult in cell studies to differentiate between the two targets, sulphydryl and disulphide, and as a sulphydryl free model of a specific type of myocrisin chemistry the globulins and platelets deserves to be studied in more in depth.

### INTERACTION OF GOLD WITH ENZYMES AND CELLS

In addition to reactions of gold(I) with thiols, disulphides and other soft ligands such as selenium and nitrogen donors, gold has a potentially important redox chemistry. Although gold(III) has a high redox value and the oxidation of gold(I) to gold(III) is not easy, it can be accomplished in the presence of hypochlorite, one of the oxidants produced by phagocytic cells. Thus, in specific sites and under the correct chemical conditions it may be possible for gold(III) to play a part in the mechanism of action (see articles by Gleichmann and Shaw). Most gold(III) compounds formed are halide or nitrogen compounds. For example, a stable gold(III) penicillamine complex is relatively easy to produce [19]. It is more difficult to produce such compounds with cysteine but the micro environments present throughout the body could lead to specific sites where gold(III) is stable. In any event, the transient formation of gold(III) could enable targeted redox reactions at donor sites of biological significance such as enzyme or transcriptase sites. Other papers in this series will develop this theme indicating that such activity does indeed occur. An additional reaction that should be borne in mind is the well known fact that in the absence of stabilising ligands such as thiols and cyanide, gold(III) and gold(I) species are very readily reduced to gold(0). For example, the reaction of gold(III) in the form of tetrachloroaurate(III) with alanine produces gold(0) colloid and acetaldehyde [2]. Gold colloid in its finely divided form, can relatively easily be re-oxidised to both gold (I) and gold(III) using sulphur compounds [2]. Thus, the precipitation of gold(0) at some point in in vitro chemistry could lead to gold(0) deposition but equally, could be a transitory species re-oxidised in the presence of oxidants such as hypochlorite.

A wide ranging survey of the action of gold compounds on enzymes indicated that many can be affected. However, there was no strong evidence that any one enzyme was selectively affected and hence would provide a possible mechanism for the clinical action of myocrisin. Glutathione peroxidase has long been suspected of being an enzyme that may be affected [20]. The selenium atom ($pK_a$ <7) at the active site of glutathione peroxidase is soft and may react with myocrisin leading to significant changes its action. This enzyme is present in a number of fractions including the plasma and the red cell cytosol. In an initial study of the activity of glutathione peroxidase following myocrisin treatment, it was found that very significant inhibition of both plasma and red cell glutathione peroxidase activity was apparent in patients treated with myocrisin compared to a group of patients being treated with NSAID's [21]. Thus, there is some in vivo rational for further studies of this enzyme. However, in-vivo glutathione peroxidase does not operate in isolation but as a component of a larger system involved in glutathione regulation. Here it must operate in concert with other enzymes such a synthase and reductase and a number of co-factors e.g. NADPH and glutathione. Thus although the clinical data suggests that gold inhibits glutathione peroxidase there may be other effects on the glutathione cycle, which affect the sulphydryl balance of the cell.

Many studies discuss the ability of myocrisin to interact directly with glutathione within biological systems and the recent paper in this volume by Tepperman et al. shows that this gold-glutathione species may form inside the cell. Glutathione reductase is yet another example of an enzyme which contains a sulphydryl residue at its active site. However, in contrast to peroxidase which has an accessible selenium binding site, it would seem that gold moieties are excluded from binding to the active site of glutathione reductase as there is little inhibition of this enzyme by either myocrisin or auranofin. This was confirmed by the use of small thiolates in the system. Mixed disulphides of glutathione with penicillamine, captopril and N-acetylcysteine are not recognised by glutathione reductase. Whereas it has long been known that glutathione peroxidase will be affected by myocrisin therapy [20], the greater impact of gold on the glutathione cycle has not been fully appreciated. The impact of down regulation of the peroxidase (GSH consumption) in the face of an active reductase (GSH production) leading to an alteration in the redox balance of the cell will be discussed below.

The gold to thiol ratio in plasma is such that the albumin thiol site cannot be saturated and it is difficult to explain the efficacy of gold on the basis of an interaction with the plasma proteins. Intracellularly, the concentration of glutathione, the major small thiol present in the cytosol is higher than that of gold by orders of magnitude. Thus, in the red cell and other cell types containing a significant concentration of glutathione, the thiol concentrations present exceed that of gold. There is no evidence at present that any one small and active thiol fraction in the cytosol is inhibited to such an extent as to suggest a unique action for gold. However, this does not apply to the cell membrane, where approximately the same number of free reactive thiols and gold atoms are present [5,8]. Since these quantities are very difficult to estimate, this calculation is liable to considerable error but it does indicate the possibility of saturation at cell membrane sites.

On the red cell membrane, the thiol groups are distributed quite widely [22] but two proteins provide the largest single concentrations of reactive thiol on the plasma (exofacial) side of the membrane of the intact cell. About 60% of the thiol groups are present on the hexose transport protein. This corresponds to two thiol groups per protein (cysteine 421 & 429) which are exposed to the plasma surface during glucose binding and transport and are thus available to bind soft metals (e.g. gold and mercury). The second largest fraction is found on the anion transporter. This protein controls water balance in the cell and chloride/carbonate exchange. This particular protein accounts for about 13% of the available reactive thiol. Again the conformation plays a significant role as to whether or not the thiol group is exposed to the plasma surface.

One method of detecting the thiol groups on the membrane is by incubation with Ellmans reagent, a disulphide. This produces a coloured anion capable of detection by electronic or Raman spectroscopy. In a clinical study of erythrocyte membrane it was possible to show that gold does bind to the exofacial surface of the cell wall [8]. The study indicates that there are at least two possible binding sites, including one on the hexose transporter. The ability to detect cysteine 421 & 429 on the transporter disappears in the presence of gold. However there is a general depression in the membrane thiol concentration which suggests that other sites are also affected. Recent studies [6, 23] indicate that in-vivo transport of gold as $[Au(CN)_2]^-$ is possible facilitating the binding of gold at a protein on the intracellular face of the membrane (spectrin?). Complexation of a metal to the functional groups at the cell wall would be expected to affect cell biochemistry via a direct modulation of transport protein activity or as a consequence of transmembrane signalling processes.

The activity of the cell surface sulphydryl functions is altered. In-vitro, erythrocytes incubated with myocrisin and treated subsequently with Ellmans reagent indicate that the activity of the membrane thiols is little affected by gold incubation [24]. However, on further incubation with Ellmans reagent, the reaction shows a clear diminution of the amount of anion resulting from exchange of the disulphide with the membrane thiol. It is clear from a number of studies that if the exofacial thiols are oxidised, the cell regenerates them . The reaction with gold treated cells suggests that the effect of the Ellmans anion is to cause a reaction involving gold bound to other thiols or disulphides deeper in the protein structure.

Treatment of red cells exofacially with thiophillic agents causes an immediate response in the cytosol. In essence, the intracellular glutathione becomes more oxidised and as the reaction continues the concentration of glutathione is reduced. These reactions are unlikely to be due to a stoichiometric reaction between glutathione and gold since the glutathione concentration is too high and the amount of gold cyanide present for transport purposes would be minimal in these in-vitro experiments. The most likely reason for such reactions is that the gold initially affects cell membrane and a transmembrane stimulus affects the thiol group. Experiments with many other thiol active compounds have since been carried out. Similar but not identical responses to intracellular glutathione have been obtained. Again the reaction is not stoichiometric and in some cases, for example with Ellmans reagent, the reagents used are believed to be cell membrane impermeant. Thus, the likelihood of a transmembrane oxidative stress created by thiols and thiophillic agents such as gold seems quite high.

The studies above are concerned with transmembrane redox balance. However, the proteins being targeted are functionally active. Our preliminary study [25] focused on the hexose transporter which is found to be subtly altered by gold. The transport of $\alpha$ and $\beta$ glucose across the membrane was followed by 13-C NMR spectroscopy. $\beta$ glucose transport involves a conformational change in the sugar transporter whereas $\alpha$ glucose does not. The rate of transport of a glucose molecule is very quick and much faster than the rate of glucose anomerisation. Thus it was difficult to measure by NMR the early stages of the kinetics but the transport of each anomer could be followed separately. That there was some control over the system was shown by the effect of the specific blocking agent cytochalasin B. In the presence of cytochalasin B the uptake of both $\alpha$-glucose and $\beta$-glucose slows down. In the presence of gold however, the transport of α-glucose would seem to be relatively unaffected whereas the transport of β-glucose is. It would seem that rather than the rate being affected gold somehow alters the equilibrium position of the nutrient balance i.e. it elevates the concentration of β-glucose. A simple explanation of this observation would be that complexation of gold to the two cysteinyl residues on the exofacial surface moderates the conformational change in the protein preventing the efflux of β-glucose. As the anomerisation reaction is slow compared to transport the effective glucose concentration inside the cell is increased. The action on a red cell is unlikely to be critical. However, similar proteins involved in other processes such as calcium regulation will be present on monocytes and other leukocytes. These proteins are required for functions particularly in situations such as the respiratory burst where considerable energy changes occur. The moderation of the action of the cell by Myocrisin could well be a key effect in vivo. Thus, the red cell is a suitable model for initial studies but further studies are required using the correct white cell fractions to discover whether incubation of gold alters transport function and whether there is a link between this transport function and immune modulation.

The studies above demonstrate the widespread distribution of gold in the cells and proteins and indicate that cell function may be altered. Plasma proteins and erythrocytes are excellent models but their function in the disease process is limited. Of more importance are the peripheral white cells which includes the monocyte. In a recent study [21], monocytes were isolated from patients treated with NSAIDs or myocrisin and assayed for hydrogen peroxide production. The levels found were higher in the gold treated patients than in controls (NSAID's and normal volunteers) but were not elevated to the extent that the cells could be classified as activated. Earlier in the discussion it was pointed out that inhibition of glutathione peroxidase in cells would lead to an increase in the amount of hydrogen peroxide in the cell and this could be a rationale for this finding. That the hydrogen peroxide levels are not at levels indicative of activation is because the other oxidant defence enzymes, such as catalase will not be affected. Confirmation of this hypothesis rests in the estimation of glutathione reductase activity. In these samples elevated reduced glutathione and depressed oxidised glutathione is observed indicative of a perturbation in glutathione regulation by gold compounds.

### CONCLUSIONS

The elucidation of the mechanism of action of gold complexes in vivo has proved an elusive target for many years. Recent developments have shown that a number of promising lines of investigation are now opening up. Not all these lines of investigation are described here since they are covered elsewhere in this series. However, they do not all point in the same direction. From a practical point of view, the main advantage of understanding the mechanism of action would be to improve the therapeutic to toxic ratio of gold compounds and to provide more selective therapy. If a significant improvement of this type could be achieved, the use of gold compounds in arthritis would become much more widespread. From this point of view, more than one effect may well be involved. For example, an understanding of a particular set of reactions which lead to a reduction in toxic side effects could well prove important.

The studies reviewed earlier indicate that any gold introduced into the body quickly becomes complexed to proteins and cells. Unless there is a labile fraction on any of these sites, the efficacy of myocrisin would depend on that small fraction of the gold which interacts with the key site in competition with the plethora of other non-active sites. Alternatively the efficacy and toxicity of gold may arise from its ability to act at a number of sites affecting the delicate balance of the biochemistry of the cell, altering cell function and cell-cell recognition mechanisms.

Of the ideas discussed in this document, two seem to have particular merit. The ability of gold to interact at functionally active transport proteins in cell membrane suggests one mechanism whereby gold can modulate the behaviour of cells. Thus, although the erythrocyte can only be regarded as a model cell type in these experiments it would seem that it is possible to alter the nutritional status of cells, for example, by altering the equilibrium position of glucose across the cell wall via complexation of gold to membrane sites. This may prepare cells for an increase in metabolism to offset additional stress associated with the oxidative pathology of the disease. The action of gold on the membrane would also seem to be important in redox signalling processes suggesting that the membrane of the cell and the proteins embedded in it are one of the key sites for gold action. For example cell fragility is a major consequence of the disease process. This is membrane effect resulting from membrane oxidation (e.g. sulphydryl functions on spectrin?) or from an imbalance in the flux of water across the cell wall (via the anion transporter?). Initial investigations of these sites indicate that these are targets for agents such as gold and small thiolates [2].

The second concept is based on the observation that the glutathione peroxidase is significantly affected by myocrisin both in vitro and in vivo. This leads to a small but significant increase in hydrogen peroxide in the system and induces an alteration in the oxidation reduction balance of the cell by elevating reduced glutathione and depleting oxidised glutathione. Gold is ineffective in acute inflammation and active in chronic inflammation. Chronic inflammation involves the accumulation of cells and interactions between cells. For example, the macrophage requires a cuff of T and B cells to be effective in chronic inflammation and the balance of T and B cell types is also a critical factor. It is becoming apparent that species such as hydrogen peroxide and the superoxide anion are used by some cells as signalling molecules [26]. One interpretation of this data is that gold modulates these signals by altering the oxidant levels in the synovium, thus altering the communication processes occurring between the macrophage, B cells and T cells. A greater understanding of the signalling mechanisms between cells based on chemical messengers like hydrogen peroxide may be critical to understand this mechanism. Effects of this type and the triggering of additional oxidant activity can both act as signalling systems and may provide a link between mechanisms involving cell membrane and those involving intracellular components.

Recent experiments on thiol compounds [27,28] offer another way of exploring gold biochemistry. Blood samples are separated to provide a monocyte fraction and red cells. The monocytes are stimulated to produce hydrogen peroxide and then recombined with the red cells in such a way that an increase in the white cell population of a factor of 10-20 is achieved. This mixture enables white cell to red cell interactions to be investigated. The white cell provides the active oxidant ($H_2O_2$) which may be involved in inflammatory responses and the red cell provides a target present in sufficient quantities for NMR signals to be recognised. The basic experiment is to induce oxidation of the erythrocyte glutathione via the interaction of oxidants produced from the active white cell. The action of gold in the system has yet to be investigated but other thiol compounds such as penicillamine, captopril and N-acetylcysteine have a very specific effect. In the absence of monocytes both penicillamine and captopril alter the redox status of the erythrocyte to a more oxidised state. However, when active monocytes are present it would seem that penicillamine and captopril protect the erythrocyte from oxidation. The erythrocyte glutathione remains substantially unchanged and the small thiols undergo sacrificial oxidation. In the presence and absence of monocytes N-acetylcysteine will induce a redox shift in the erythrocytes to a more reduced state. This is because N-acetylcysteine has two functions in this system. One which alters the redox balance of the erythrocyte to a more reduced state and one which switches off active monocytes stemming the flow of oxidants. Although these species are all thiols they have substantially different effects on the cell and more importantly on cell cell interactions.

Thus, at the present moment a number of different ideas are being pursued in different places and it is to be hoped these activities will lead to a more definitive understanding of the mechanism of action of gold.

### REFERENCES

1. J.W.Sigler, B.G.Bluhm, H.Duncan, J.T.Sharp, D.C.Ensing and W.R.McCrum *Arthritis Rheum.* **16**, 756 (1972)
2. W.E.Smith and J.Reglinski *Presp. Bioinorg. Chem.* **1**, 183 (1991)
3. A.McNeilly PhD thesis Strathclyde University 1980
4. C.F.Shaw, N.A.Schaeffer, R.C.Elder, M.K.Eidness, J.M.Trooster and G.H.M.Calis *J. Amer. Chem. Soc.* **106**, 1311 (1984)
5. J.Egila, D.Littlejohn, W.E.Smith and R.D.Sturrock *J. Pharm. Biomed. Anal.* **10**, 639 (1992)
6. G.G.Graham, T.M.Havisto, H.M.Jones and G.D.Champion *Biochem. Pharmacol.* **33**, 1257 (1987)
7. K.Tepperman *Proc. 3rd Intml. Conf. Au /Ag Med. in Metal Based Drugs*, this issue.
8. J.M.Campbell, J.Reglinski, W.E.Smith, D.Porter and R.D.Sturrock *Ann. Rheum. Dis.* **51**, 969 (1992)
9. J.Campbell PhD thesis Strathclyde University (1993)
10. R.H.Persellin and M.Ziff *Athritis Rheum* **9**, 57 (1966)
11. R.C.Elder and M.K.Eidsness *Chem Rev.* **87**, 1027 (1987)
12. D.H.Brown, G.C.McKinley and W.E.Smith *J. Chem. Soc. Dalton Trans* 1874 (1977)
13. C.McDonald, P.Mortimer, G.McAllister, P.E.Gardiner, J.Reglinski and W.E.Smith *J. Pharm. Biomed Anal* in Press Ms NoF94-19 (1994)
14. M.T.Coffer, C.F.Shaw, A.L.Hormann, C.K.Mirabelli and S.T.Crooke *J. Inorg. Biochem* **30**, 177, (1987)
15. A.A.Isab, A.L.Hormann, M.T.Coffer and C.F.Shaw *J. Amer. Chem. Soc.* **110**, 3278, (1988)
16. J.Reglinski, S.Hoey and W.E.Smith *Inorg. Chim. Acta.* **152**, 261 (1988)
17. P.Mortimer, J.Reglinski and W.E.Smith unpublished results

18. K.M.S.McKauge, C.J.L.Lock, F.McCrae, W.E.Smith, H.Buchanan, W.F.Kean and J.Reglinski *J. Pharm. Sci.* **82**, 174, (1993)
19. D.H.Brown, G.McKinlay and W.E.Smith J. Chem. Soc. Dalton Trans. 562, (1978)
20. J.Chaudiere and Al.Tappel *J. Inorg. Biochem.* **20**, 313 (1980)
21. Unpublished data, J.Reglinski
22. C.Chilles, M.Mulheron, F.McCrae, J.Reglinski, W.E.Smith, M.Brezski and R.D.Sturrock *Ann. Rhuem. Dis.* **49**, 668, (1990)
23. R.C.Elder, Z.Zhao, Y.Zhang, J.G.Dorsey, E.V.Hess and K.Tepperman *J. Rheumatol.* **20**, 268 (1993)
24. W.E.Smith, J.Reglinski, S.Hoey, D.H.Brown and R.D.Sturrock *Inorg. Chem.* **29**, 5190 (1990)
25. P.E.McGowan, J.M.Campbell, J.Reglinski and W.E.Smith *Inorg. Chim. Acta.* **210**, 1 (1993)
26. R.H.Burdon and V.Gill *Free Radical Comm.* **19**, 203 (1993)
27. P.E.McGowan, J.Reglinski, W.E.Smith, R.Wilson and R.D.Sturrock *FEBS Lett.* **314**, 455 (1992)
28. J.Russell PhD Thesis Strathclyde University (1994)

